# Multiple Lenses to Unearth Hidden Voices: Living with Diabetic Foot Ulceration in an Afro-Caribbean Community

**DOI:** 10.3390/ijerph22020304

**Published:** 2025-02-18

**Authors:** Laura Lovell, Michael H. Campbell, Natalie Greaves

**Affiliations:** Faculty of Medical Sciences, University of the West Indies Cave Hill Campus, Bridgetown BB11000, Barbados; michael.campbell@cavehill.uwi.edu (M.H.C.); natalie.greaves@cavehill.uwi.edu (N.G.)

**Keywords:** diabetic foot, diabetes, diabetic foot ulcer, Afro-Caribbean, foot ulcer

## Abstract

(1) Background: This study was conducted in the small island developing state of Barbados, which has dubiously earned the title of “amputation capital of the world”, to understand perspectives of persons living with diabetic foot ulceration. (2) Methods: An exploratory multi-lens approach was used (focus groups; dyads; and triads) to gather indigenous Afro-Caribbean perspectives of living with diabetic foot ulceration that may be obscured by using a single method. (3) Results: Findings in this group highlighted the necessity of creating culturally sensitive education tools, as well as understanding how mistrust of local health systems may play a role in decisions to delay seeking health services despite ease of access with no cost at point of care. Problematic historical relationships with health systems among Afro-Caribbean people, for whom oral traditions motivate preference for traditional medicines instead of Western/colonial treatments from North America or Europe, may be deeply entrenched in this population and contribute to health beliefs and behaviors. (4) Conclusions: This paper addresses the gap in the literature regarding the use of qualitative methodologies to explore the beliefs of Afro-Caribbean people within their native context to inform design of culturally responsive self-education programs.

## 1. Introduction

The International Working Group on the Diabetic Foot (IWGDF) defines diabetes-related foot disease (diabetic foot) as “disease of the foot of a person with current or previously diagnosed diabetes mellitus which may include one or more of the following: peripheral arterial disease, infection, gangrene, amputation, ulcer(s), or peripheral neuropathy” [1]. Diabetic foot and related complications present a significant health systems burden due to the overwhelming costs of outpatient dressings as well as protracted in-hospital stays. In a 2014 study, diabetic foot disease (DFD) was found to contribute to 30% of hospital admissions at Barbados’ lone public hospital; these represented 89% of all diabetes-related admissions [2]. Diabetic foot ulceration (DFU) is a “break in the skin of the foot that includes minimally the epidermis and part of the dermis in a person living with diabetes” [1]. Ischemia and infection are the main indications for admission related to complications of diabetic foot [3], and management requires a multidisciplinary approach with vascular interventions; local and systemic infection management; surgical interventions; and likely outpatient follow-up in wound care and orthotic clinics [4].

### 1.1. Diabetic Foot in Barbados

Epidemiological data support the unenviable characterization of Barbados as the “amputation capital of the world” [5,6]. Various educational efforts and attempts to improve the diabetic foot landscape have met with seemingly little success. As a small island developing state with a high non-communicable disease (NCD) burden, where a quarter of adults are diagnosed with an NCD [7], Barbados is on the frontline of NCD-related public health challenges, including those presented by DFD. As early as 1998, the burden of DFD in the sole public tertiary hospital accounted for 67.5% of admitted surgical patients over a six-month period [8]. These patients too frequently required major amputations for “many trivial preventable problems” [8]. A recent study of DFD in a Barbadian primary care diabetes specialist clinic setting found a 1-year prevalence of 14.7% for diabetic foot ulceration, with associated complications of retinopathy, antiplatelet therapy, and chronic kidney disease noted [9]. DFD education and amputation prevention programs have included the World Diabetes Foundation Step-by-Step program, which trained over 200 physicians and nurses in Barbados and St Lucia from 2009 to 2012 [10]. Unfortunately, these efforts have not realized lower rates of foot ulceration or limb loss in the decade since conclusion of the intervention [11].

### 1.2. Patient Populations and Barriers and Facilitators to DFU

Barriers and facilitators to effective DFU treatment have been explored in various populations, but evidence for Afro-Caribbean people is limited. A 2023 qualitative systematic review and meta-analysis exploring perceptions and experiences of persons with diabetes towards DFU highlighted four overarching themes: perceptions of DFUs (realization and reasons), coping with DFU (including persons’ behaviors towards treatment and management and perceptions towards amputation), expectations (expectation of health personnel and future expectation), and living with DFU (physical and emotion burdens, economic burdens, and change in life) [12]. Diasporic Afro-Caribbean populations in the United Kingdom living with type 2 diabetes have noted mistrust in medical systems and preference for natural remedies to traditional medical techniques as barriers to treatment [13]. Prior explorations within a rural Barbadian population found that ethno-botanical practices were present in 75% of the population with its use linked to demographic variables of education and health insurance [14]. These are echoed in qualitative studies emerging in Southeast Asia, the United Kingdom, mainland Europe, and the United States [15,16,17], but research on groups in the Caribbean region specifically surrounding diabetic foot disease is lacking.

Although disparities in outcomes for Afro-Caribbean people with DFU are well established [18], the barriers and facilitators for this population remain poorly understood. Afro-Caribbean people represent the predominant ethnic group in Barbados, which is also burdened by high DFU rates. Better understanding of the experiences of Barbadian patients with DFU has important implications for diabetes care in both Barbados and other communities with similar populations.

## 2. Materials and Methods

Paradigm, methods and ethical approval: A qualitative methodology was selected given the exploratory, hypothesis-generating nature of the work and the need to generate rich contextual descriptive data to explore the factors influencing the lived experiences of the participants with a stigmatizing medical condition [19,20]. The work was undertaken using a qualitative methodology with an interpretivist stance, which generated meaningful participant accounts of their lived experiences and perceptions of DFU as a person living with diabetes (PLWD) using focus groups and dyads [20,21]. Ethical approval was obtained from the University of the West Indies/Barbados [22].

### 2.1. Setting

Barbados is an Eastern Caribbean country with a lone public hospital receiving patients from both private and public referral sources. Public health care is free of cost for all residents at the point of care and is funded primarily through taxation [23]. Primary public health care for diabetes is facilitated by local polyclinics or primary health centers, as well as one multidisciplinary center for diabetes. Patients who are newly diagnosed, uncontrolled, or have active foot ulceration are referred to the center for a six-month period of care [9].

### 2.2. Sampling Strategy and Participants

Participants were recruited using purposive sampling at two rural and two urban/suburban polyclinics in addition to the multidisciplinary diabetes center wound care clinics, which bridge primary and secondary care. High-risk and complex wound patients are currently managed within the hospital wound clinic; only clinically stable patients attend polyclinics or the multidisciplinary clinic. A total of five groups, including focus groups, triads, and dyads, were included in this study. The inclusion criteria are seen in Table 1 below.

### 2.3. Data Collection

The researcher (LL), who is deeply embedded in the field of diabetic foot care in Barbados, developed the interview guide informed by theories of planned behavior [24] and the need to develop culturally appropriate foot programs and educational tools to more adequately address barriers to care [24,25,26]. The focus group guide explored three major areas: diabetic foot care knowledge, the Barbados experience of diabetic foot, and amputation and limb loss. The interview guide without sub-questions is seen in Table 2 below.

Focus groups took place from June 2023 to February 2024 and were conducted by LL with debriefing with NG afterwards. Field notes and contact summary sheets were also developed after each interview. Data collection was complete when no new emerging themes arose from the datasets (i.e., when saturation was achieved). The data collection for the participants with DFU was therefore concluded at *n* = 15 from a total of five sites.

### 2.4. Analytical Approach: Secondary Analysis

All interviews were transcribed modified verbatim by LL (a postgraduate-trained qualitative researcher). Two interviews were then independent double-coded manually by LL and NG (a doctoral-level qualitative researcher) to ensure consistent coding and data interpretation with any differences resolved through discussion [25]. The initial coding list of 29 codes was then applied to transcripts using Atlas.ti version 23 to aid in data reduction. The basic themes were then determined through coding frequencies, analyzing patterns in co-occurrences in Atlas.ti version 23 software. The emerging data revealed four organizing themes and one global theme as shown in Table 3.

## 3. Results

The recruitment allowed for participation of five groups with fifteen participants. Table 4 summarizes the participant profiles.

Site Key:

1. Multidisciplinary Center
2. Urban/Suburban Facility
3. Rural Facility
4. Rural Facility
5. Urban Facility

### 3.1. Data Condensation

Inductive coding through the 29-item framework identified the basic themes of “culturally accepted treatment as a barrier to evidenced based care”, “the information desert”, “emotional impact of DFU and limb loss”, and “private/public health systems”. The Sankey diagram in Figure S1 is a further presentation of the findings. Further condensation identified the organizing themes (OT) of Information, Disparity, and Feelings that we subsequently used to present the findings.

The interconnection of these organizing themes leads to a systemic failure to empower individuals living with DFU to achieve remission and ultimately improved quality of life. The further discussion of this web of relationships and its implications for health systems, bounded by principles of empathic neutrality and reflexivity, produced the global theme of Health Care-Related Stigma and Disenfranchisement. These results are now presented using OTs.

### 3.2. OT1: Information

#### 3.2.1. Sources of Trusted Information on DFU

This theme captured the perceived lack of trusted information available on DFU as well as the complex ways that participants see information as trusted. Reliance on doctors was in keeping with paternalistic patterns.

“*The best information would come from the doctor or the nurse*”

**P1, FG 3, 58 years, male**

However, some participants preferred to obtain information from the “neighbor”, seen as anyone within their community who was providing information at the time who they viewed as a trusted source:

“*One or two people from my neighbourhood (told me about my foot)… If I go to the shop or anything so would be like man…. you foot look funny boy! You diabetic you know be careful before you lose it. I frightened when they say I would lose it*.”

**P2-2, FG4, 56-year-old male**

#### 3.2.2. Caribbean Traditional Remedies

Traditional therapies for healing of DFU still persist among participants, with some preferring local remedies to heal DFU as opposed to presenting to a medical facility for initial care. These treatments appear well known amongst the community and no doubt have been passed down through generations by oral traditions, but they often contradict the information advised in Western medical approaches.

*“1: Well, the same fella that I now tell you about…he would have tried saltfish skin and all sort of things and it just made the foot worse*

*3: Yes, that is the thing (remedy) for nail juk (puncture)”*

***Exchange between P1 and P3 from FG2 (58-year-old male and 69-year-old female)***

*“My wife saying now she going to get some grated paw-paw (papaya) and see if that would heal it…”*

**P1, FG3 (58-year-old male)**

#### 3.2.3. Information Sources

Diabetic foot education shared via print media was not a preferred method of transfer of information according to participants. Identified barriers of illiteracy, which may not be readily communicated to medical personnel, gave rise to preferences for videos, one-to-one education sessions, and talk programs as opposed to print.

*“I think we need more people more educated in this thing….; remember that a lot of people cannot read! So, if persons would come out and speak to them, they would understand ……. That is what people don’t know”*

**P2-1, FG4 67-year-old female**

Lack of provision of information by healthcare professionals was also seen as a challenge among participants, and they believed they were ill-equipped with the tools for self-management.

*“We should (know about diabetic foot) but I never find out… anything…. the source of the diabetic foot no one ever told me….neither here at the clinic or my private doctor”* 

**P2, FG3, 79-year-old female**

### 3.3. OT2: Disparity

The differences in care between private versus primary care in Barbados are a perceived barrier in the management of DFU among the Barbadian population. Seen by participants as a place where you receive superior treatment if you have “money”, the private sector for DFU appeared to be viewed as the better option compared to the public sector, where the quality of care was viewed as superior. The participants spoke of experiencing feeling vulnerable in polyclinics and some participants did not appear to easily trust staff.

#### 3.3.1. Vulnerability of Users in Public Systems

Long wait times and discrimination towards some users of the public healthcare system were some of the concerns that users of the public system expressed. Especially for those working, wait times proved discouraging, as these related to loss of income, further increasing the vulnerability of patients.

LL: “*because you were saying that you saw people from off the streets, people from everywhere, … in foot clinic….*”

P4, FG4: “*You are exposed to all infections in there (hospital dressing clinic)*”

**Exchange between interviewer and P4, FG1 a 76-year-old female**

“*2: Even when you go to clinic…. you hear…man where you going….you know them people does make your foot get chop off?*

*LL: That’s the word… (is that what people are saying in the community?)*

*2: Yep!!*

*1: Yea yea…*

*2: Meaning that at the clinic you don’t get the proper attention…and eventually you get you foot amputated.”*

**Exchange between P1 and P2 in FG3 and the interviewer**

#### 3.3.2. Lack of Trust in Public Primary Care Staff

Participants also expressed that they perceived a lack of confidentiality amongst healthcare staff. They felt their medical condition may be discussed with non-medical personnel.

“*We telling the truth! Clinic B too… When I used to go to Clinic B, I had a friend who used to work there and every day when she come home… “you know she got a bad foot” you know this, you know that*”

**P2, FG1, 62-year-old female**

“*They (are) not confidential…the nurses does talk.*”

**P3, FG1, 50-year-old female**

### 3.4. OT3: Feelings

#### 3.4.1. Fear of the Hospital and Limb Loss

Hospital perceptions tied to fear of the facility were seen to be a major cause of limb loss. Participants expressed that limbs were amputated by doctors for payment as common practice and that surgeons found it easier to cut off a limb rather than attempt limb salvage.

“*P2: Even when you go to clinic…. you hear…man where you going….you know them people does make your foot get chop off?*”

**P2, FG3, 79-year-old female**

“*LL: What have you heard that makes you scared?*

*P1: Everything! Them is cut! Cut! Cut! Cut!”*

**P1, FG 4, 52-year-old male**

“*Well, I would tell you the truth. If I go to hospital and they tell me to take off a foot or a limb I would be hesitant. They say you getting paid to do that but I don’t know that. I hear that the doctors are paid to cut off your foot and your hand but especially your foot*”

**P1, FG2, 69-year-old female**

#### 3.4.2. Global Theme: Health Care-Related Stigma and Disenfranchisement

**Figure 1:**
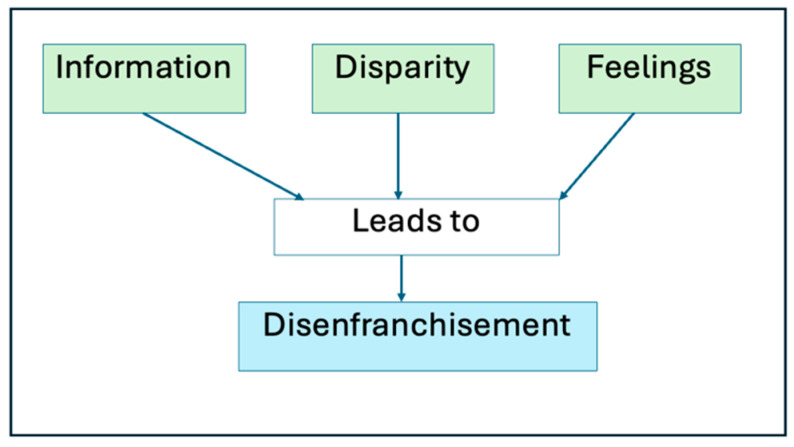
The Relationship between Information, Disparity, and Feeling and Resultant Disenfranchisement.

The overarching/global theme for the research was health care-related stigma and disenfranchisement. This theme encapsulated the three organizing themes derived and explains how health system challenges and perceived barriers interact. An information desert among participants coupled with feelings of fear and mistrust in the public primary healthcare systems, also fed by perceived differences in public and primary healthcare, contributed to the possible increased perception of health care-related stigma (felt, perceived, and possibly infrastructural) and subsequent disenfranchisement of individuals who were then vulnerable to limb loss. These processes are represented in Figure 1.

### 3.5. Reflexivity Statement

The researcher LL is aware of their professional role and position within the landscape of DFU management in Barbados. NG is a medical professional currently practicing within the climate of non-communicable diseases in Barbados. To minimize the risk of bias, LL created summary sheets and used these along with self-reflection as data emerged, as part of the process of internal and external accountability to ensure the truest reflection of the participants’ voices within the presented account.

## 4. Discussion

Barbados has a history of colonialism and class structure where the voice of marginalized groups that routinely access social services is underrepresented. This, however, can be unearthed using multiple lenses of qualitative data collection through focus groups, dyads, and triads to layer the dynamics of intersection of participants and their environments. This study sought to enlighten the narrative surrounding the complications of DFU among persons living in this Caribbean country, whose experiences and voices have previously been underrepresented in documented research findings. Participants also recounted how societal knowledge of the “amputation capital of the world” colored the backdrop for their perceptions and beliefs.

### 4.1. OT1: Information

Trusted sources of information identified by participants appeared varied. While participants valued information provided by medical personnel, information from “the neighbor” or trusted persons within their community sometimes took precedence. Treatments and remedies described in oral traditions seemed to be especially relevant and often preferred, as is seen in other Caribbean societies with prevalent “substitution culture” [27]. “Substitution culture” was a term initially coined by Cawich et al. to describe the preference for ethno-botanical medicines or any alternative medical treatment instead of standard medical care [27]. For receiving information, participants indicated preference for one-to-one approaches to education that provide opportunity for discussion. Public information programs on television and radio and videos were preferred over written material. These findings may be useful for planning and developing educational programs for diabetic foot education and suggest that storytelling techniques and videos may be more effective promotion methods.

These findings appear to follow on from those identified in a 2015 study in a Barbadian population where participants (persons living with diabetes) described heavy reliance on “self-care” abilities and reported difficulty navigating a public health system with scarce availability of footcare appointments [26]. Without effective educational resources, patients are left vulnerable and unsure of what diabetic footcare that minimizes complications looks like. There may also be hidden narratives of class and stigma that may be impacting the healthcare experience.

This situation is not dissimilar to those in other diasporic populations, including inner-London black African and Caribbean communities where “getting the message across” is a challenge of diabetes self-management education [27,28]. Culturally informed education programs responsive to the needs of Caribbean populations are essential for developing programs to engage patients in the design of true patient-centered care.

### 4.2. OT2: Disparity

Unlike the situations in the United Kingdom and United States, where people of African descent are an ethnic minority, Afro-Caribbean people are the majority ethnic group in Barbados. The long-standing cultural distrust of medical systems and transgenerational preference for alternative treatments seem to be less linked to ethnic minority status and perhaps more to community experiences and preferences for natural or alternative therapies. This has been previously documented in studies reflecting on diabetic education [28], asthma, smoking and lung cancer [29].

“Socialized Medicine” through a Beveridge model of healthcare provides healthcare free to all its citizens at the point of care through tax payments [30]. Within Barbados, this model exists but, despite the availability of all major specialty services for medical care at no cost at point of care to all citizens, participants viewed public primary healthcare as inferior to private primary healthcare. Long wait times and a lack of confidentiality among clinic staff were seen as concerning to some participants. This reflected some distrust for the system, which was also viewed by participants as resorting to amputations prematurely.

The lack of trust in evidence-based medical practices and health systems links to poor cultural compatibility with health education within the current context. Cultural identity needs to be a factor in developing health education programs as it is critical to meeting the needs of users, especially within Afro-Caribbean communities.

### 4.3. OT3: Feelings

The overwhelming feelings of negativity surrounding the risk and experience of limb loss were captured by many of the participants’ accounts. Perceptions of hospital care, especially surrounding referral if needed for tertiary care of DFU, were linked to perceptions of automatic amputation if referred. This was mainly influenced by community beliefs and not lived experiences and was even colored by some HCPs, who indicated that patients recounted perceived high rates of amputation and mortality linked with DFU outcome. This is not verified by any official source, but has been trickled through the community and has become synonymous with DFU in Barbados. This may be a barrier to seeking care early, especially when culturally promoted alternative therapies are seen as effective as well. Persons may therefore turn to these alternative and folk remedies before seeking medical attention, as fear of hospital referral and associated likelihood of amputation are powerful disincentives.

### 4.4. Limitations

Our study was primarily carried out in public polyclinics and a multidisciplinary center in Barbados, and the study population comprised mainly older persons who were not dual users of private and public systems. As a result, views of private health system users were not included. However, thematic saturation was reached among all the participant sites, and this adds to the robustness of the dataset.

Despite not including private health system users, this study adds to the understanding of persons who are users of the same social supports that can be strengthened in primary care to provide improved tertiary care outcomes. Their voices can contribute meaningfully to the creation of culturally responsive education tools for Afro-Caribbean people.

## 5. Conclusions

Healthcare disparities among Afro-Caribbean populations exist not only in diasporic communities but also in communities of origin and may be due to access and utilization. As a result, healthcare systems should evolve to embrace ingrained cultural healthcare understandings which may not always align with the healthcare provision and education tools of the global north. HCP engagement with African and African diaspora communities should consider cultural attitudes beliefs and seek to engage patients with relatable educational interventions. As “the neighbor” within their communities is seen as a trust figure, communication through community participatory involvement and non-didactic approaches to education can help build trust in health systems.

Malalignment of patient and healthcare practitioner beliefs increases risk of non-adherence and various footcare behaviors that contribute to DFD [27]. This same study found that motivation for footcare was derived from lived experiences rather than simply from traditional patient education [27]. So-called “substitution culture” and the use of alternative therapies for management of DFD is a Caribbean phenomenon documented in neighboring countries [27] and anecdotally known to exist in Barbados. As a result, evidence-based medicine and traditional approaches for patient education must involve the lived experiences of patients to develop culturally appropriate tools for addressing barriers and facilitators of DFD.

The exploration of the lived experience of diabetic foot ulcer in a SIDS highlights cultural barriers and opportunities for development of patient-centered diabetic foot education programs which can empower indigenous and diasporic Caribbean communities at risk of foot ulceration.

## Figures and Tables

**Table 1:** Inclusion Criteria of Participants.

Inclusion Criteria
- PLWD with active diabetic foot ulcer
- Medical capacity to provide consent
- Age > 18 years

**Table 2:** Focus Group Guide.

Exploring diabetic foot care knowledge	What do you understand by the term diabetic foot (ulcer)?
	How do you think people get information about diabetic foot?
	What types of ways do you think people should get information on diabetic foot?
	What types of things do you think persons should do if they have a foot problem and are living with diabetes?
	If someone living with diabetes were referred to the hospital, do you think they should go? When do you think seeking care at the hospital is necessary?
Exploring the Barbados Diabetic Foot	Is there a problem with diabetic foot in Barbados?
	What sorts of healthcare persons are involved in diabetic foot care in Barbados?
	What sorts of things do you believe cause persons to have diabetic foot ulcers?
	Why do you think people lose limbs who have diabetes?
	What do you believe can improve the outcome of persons who have ulcers for limb loss (prevent limb loss in diabetic foot)?
Amputation and Limb Loss	Can you think of any ways in which amputations (loss of toes or foot) could be prevented?
	What would you say can be used to decrease the level of amputations in Barbados?

**Table 3:** Code Names and Basic Themes Derived.

No.	Code Name and Abbreviations	Code Meaning: All References by Participants Relating to the following	Basic Themes
1	Neighbor (neib)	References to choice of information from non-medical personnel rather than from medical personnel or approved sources	Culturally acceptable treatments as a barrier to EBM Care
2	Evidence-Based Medicine (ebm_m)	References to the use of evidence-based management/guidelines for the treatment of diabetic foot by healthcare professional (HP)	
3	Reliable sources of information (reli)	Reliable sources of information as perceived by HP or client	
4	Alternative Medicine (alt_m)	Alternative management practice compared to/along with standard of care (evidence-based medicine) as a treatment option for DFU	
5	Foot Behavior (behav_foot)	Behavior modification observed in patients with DFU Barbados	Information Desert
6	Limb Loss (cause_ll)	Causes of limb loss	
7	Diabetic Foot Ulceration (causes_Dfu)	Causes of DFU	
8	Define (defn)	Individual understanding of diabetic foot ulcer definitions	
9	Program (progt)	Reference to program types/methods for information dissemination on DFU	
10	Information sources (info_C)	Information sources on diabetic foot ulcers patients seek	
11	Initial HCP (initial)	The initial care provider for management of the acute DFU	
12	Information HCP (info_p)	Health providers providing information on diabetic foot	
13	Responsibility HP (resp)	Responsibility of the HP to provide information on diabetic foot	
14	Dietary associations (diet_dfu)	The importance of diet in diabetic foot ulcers	
15	Initial Presenting Sign (ini_p)	Key clinical signs that persons (patients/clients) use to indicate or trigger the initiation of seeking health care intervention for diabetic foot	
16	Lack of Information (lackinf)	References to the experienced and perceived lack of information on diabetic foot ulcer to/for the clients	
17	Responsibility Pt (resp_p)	Any reference that suggests that responsibility for seeking information on DFU or limb loss is for a PLWD to seek out	
18	Role of HCP (healpract_team)	Role and responsibility of members of the diabetic foot team (HCP)	
19	DFU education (dfu_edu)	What diabetic foot ulcer education may or should look like (ideal scenario)	
20	Health-seeking Fear (fear_h)	Fear of seeking care at the hospital or clinic for foot complaint	Emotional Impact of DFU and Limb Loss
21	Emotions (emotion_Dfu)	Participant’s emotion due to DFU or limb loss	
22	Spirituality (spiri)t	The spiritual connection with diabetic foot ulcer or limb loss	
23	Hospital Perceptions (hosp_perc)	Hospital (local and other) experiences and perceptions	
24	Public Healthcare (moh_pol)	Policies on diabetic foot in public healthcare settings	Public/Private Health Systems
25	Foot Problem (probfoot)	Participant’s perception of DFU being a significant/priority health condition in Barbados	
26	Public-Private Differences (pub_pr)	Differences in public and private healthcare systems	
27	Public Policy (pub_r)	The role of the public health system in the management of diabetic foot	
28	Patient Resources (resour_pts)	Challenge of socially disadvantaged status in role in DFU	
29	System Resources (resource)	Challenges with (health care setting) resources to adequately manage and screen diabetic foot	

**Table 4:** Participant profiles.

Site	Participant Number	Duration of Disease (years)	Sex	Age	DFU Duration	Occupation
1	P1	>10 years	M	73	>10 years	Livestock farmer
	P2	>10 years	F	62	2 years	Housekeeper
	P3	>10 years	M	50	>5 years	Medically Unfit
	P4	>10 years	F	76	>5 years	Retired administrative worker
2	P1	>10 years	M	58	6 months	Self-employed tradesman
	P2	3 years	F	69	3 years	Retired
	P3	>10 years	F	54	1 month	Retired
3	P1	>10 years	M	58	6 months	Unemployed
	P2	>10 years	F	79	>20 years	Retired
4	P1-1	>10 years	F	71	1 year	Retired sales clerk
	P2-1	>10 years	F	67	6–9 months	Retired housekeeper
	P1-2	>10 years	M	56	1 year	Agricultural worker
	P2-2	>10 years	M	52	1 year	Fisherman
5	P1	<1 year	F	54	<3 months	General worker
	P2	>5 years	F	58	<3 months	Unemployed

## Data Availability

The data presented in this study are available on request from the corresponding author due to privacy.

## Supplementary Materials

The following information can be downloaded at: https://www.mdpi.com/article/10.3390/ijerph22020304/s1. Figure S1: Sankey Diagram with Key for Code Descriptions.

## References

[1] Van Netten J.J., Bus S.A., Apelqvist J., Chen P., Chuter V., Fitridge R., Game F., Hinchliffe R.J., Lazzarini P.A., Mills J. (2023). Definitions and criteria for diabetes-related foot disease (IWGDF 2023 update). Diabetes Metab. Res..

[2] Taylor C.G., Krimholtz M., Belgrave K.C., Hambleton I., George C.N., Rayman G. (2014). The extensive inpatient burden of diabetes and diabetes-related foot disease in Barbados. Clin. Med..

[3] Da Ros R., Assaloni R., Michelli A., Brunato B., Barro E., Meloni M., Miranda C. (2024). Burden of Infected Diabetic Foot Ulcers on Hospital Admissions and Costs in a Third-Level Center. Diabetology.

[4] Musuuza J., Sutherland B.L., Kurter S., Balasubramanian P., Bartels C.M., Brennan M.B. (2020). A systematic review of multidisciplinary teams to reduce major amputations for patients with diabetic foot ulcers. J. Vasc. Surg..

[5] Hambleton I.R., Jonnalagadda R., Davis C.R., Fraser H.S., Chaturvedi N., Hennis A.J. (2009). All-cause mortality after diabetes-related amputation in Barbados: A prospective case-control study. Diabetes Care.

[6] Hennis A.J.M., Fraser H.S., Jonnalagadda R., Fuller J., Chaturvedi N. (2004). Explanations for the high risk of diabetes-related amputation in a Caribbean population of black african descent and potential for prevention. Diabetes Care.

[7] Springer R.A., Elliott S.J. (2019). “There’s Not Really Much Consideration Given to the Effect of the Climate on NCDs”-Exploration of Knowledge and Attitudes of Health Professionals on a Climate Change-NCD Connection in Barbados. Int. J. Environ. Res. Public Health.

[8] Walrond E.R., Ramesh J. (1998). Quality of care of patients with diabetic foot problems in Barbados. West Indian Med. J..

[9] Lovell L., Dunkley A., Webb D., Jarvis J., Gillies C. (2022). Incidence, prevalence, and potential risk factors for diabetic foot ulceration: A retrospective review at a multidisciplinary centre in Barbados. Int. Wound J..

[10] World Diabetes Foundation Step-by-Step Foot Care Training. https://www.worlddiabetesfoundation.org/what-we-do/projects/wdf09-0438/.

[11] Bokinni Y. (2024). Barbados is in the grip of a diabetic foot amputation crisis. BMJ.

[12] Ma L., Chen J., Sun Y., Feng Y., Yuan L., Ran X. (2023). The perceptions of living with diabetic foot ulcers: A systematic review and meta-synthesis of qualitative studies. J. Tissue Viability.

[13] Brown K., Avis M., Hubbard M. (2007). Health beliefs of African-Caribbean people with type 2 diabetes: A qualitative study. Br. J. Gen. Pract..

[14] Vujicic T., Cohall D. (2021). Knowledge, Attitudes and Practices on the Use of Botanical Medicines in a Rural Caribbean Territory. Front. Pharmacol..

[15] Hill A., Ellis M., Gillison F. (2022). Qualitative exploration of patient and healthcare professional perspectives on barriers and facilitators to foot self-care behaviors in diabetes. BMJ Open Diabetes Res. Care.

[16] Semerci Çakmak V., Çetinkaya Özdemir S. (2024). Patients with diabetic foot ulcers: A qualitative study of patient knowledge, experience, and encountered obstacles. J. Tissue Viability.

[17] Sari Y., Yusuf S., Haryanto H., Sumeru A., Saryono S. (2022). The barriers and facilitators of foot care practices in diabetic patients in Indonesia: A qualitative study. Nurs. Open.

[18] Mcdermott K., Fang M., Boulton A.J.M., Selvin E., Hicks C.W. (2024). Etiology, Epidemiology, and Disparities in the Burden of Diabetic Foot Ulcers. Diabetes Care.

[19] Denzin N.K., Lincoln Y.S. (2013). The Landscape of Qualitative Research.

[20] Green J., Thorogood N. (2018). Qualitative Methods for Health Research.

[21] Morgan D.L., Ataie J., Carder P., Hoffman K. (2013). Introducing Dyadic Interviews as a Method for Collecting Qualitative Data. Qual. Health Res..

[22] World Medical Association WMA Declaration of Helsinki—Ethical Principles for Medical Research Involving Human Subjects. https://www.wma.net/policies-post/wma-declaration-of-helsinki-ethical-principles-for-medical-research-involving-human-subjects/.

[23] Ministry of Health and Wellness Health Financing. https://www.health.gov.bb/About/Health-Financing.

[24] Coreil J. (2010). Social and Behavioral Foundations of Public Health.

[25] Hinds J., Greaves N., Harewood H. (2023). Diabetes self-management and social support during the COVID-19 pandemic: Perspectives of older adults living in Barbados. Dialogues Health.

[26] Guell C., Unwin N. (2015). Barriers to diabetic foot care in a developing country with a high incidence of diabetes related amputations: An exploratory qualitative interview study. BMC Health Serv. Res..

[27] Cawich S.O., Naraynsingh V., Jonallagadda R., Wilkinson C. (2019). Caribbean “substitution culture” is a barrier to effective treatment of persons with diabetic foot infections. World J. Surg. Proced..

[28] Goff L.M., Moore A., Harding S., Rivas C. (2020). Providing culturally sensitive diabetes self-management education and support for black African and Caribbean communities: A qualitative exploration of the challenges experienced by healthcare practitioners in inner London. BMJ Open Diabetes Res. Care.

[29] George M. (2012). Health beliefs, treatment preferences and complementary and alternative medicine for asthma, smoking and lung cancer self-management in diverse Black communities. Patient Educ. Couns..

[30] Wallace L.S. (2013). A view of health care around the world. Ann. Fam. Med..

