# Correction: Lovell et al. Multiple Lenses to Unearth Hidden Voices: Living with Diabetic Foot Ulceration in an Afro-Caribbean Community. *Int. J. Environ. Res. Public Health* 2025, *22*, 304

**DOI:** 10.3390/ijerph22060847

**Published:** 2025-05-29

**Authors:** Laura Lovell, Michael H. Campbell, Natalie Greaves

**Affiliations:** Faculty of Medical Sciences, University of the West Indies Cave Hill Campus, Bridgetown BB11000, Barbados; michael.campbell@cavehill.uwi.edu (M.H.C.); natalie.greaves@cavehill.uwi.edu (N.G.)

The authors have requested that the following changes be made to the original publication [1], Vujicic and Cohall (2021) and George (2012) were not cited [2,3], Brown et al (2007) was not cited in the correct location [4]. The citations have now been inserted and corrected.

Section 1.2., paragraph 1 and should read:

Barriers and facilitators to effective DFU treatment have been explored in various populations, but evidence for Afro-Caribbean people is limited. A 2023 qualitative systematic review and meta-analysis exploring perceptions and experiences of persons with diabetes towards DFU highlighted four overarching themes: perceptions of DFUs (realization and reasons), coping with DFU (including persons’ behaviors towards treatment and management and perceptions towards amputation), expectations (expectation of health personnel and future expectation), and living with DFU (physical and emotion burdens, economic burdens, and change in life) [12]. Diasporic Afro-Caribbean populations in the United Kingdom living with type 2 diabetes have noted mistrust in medical systems and preference for natural remedies to traditional medical techniques as barriers to treatment [13]. Prior explorations within a rural Barbadian population found that ethno-botanical practices were present in 75% of the population with its use linked to demographic variables of education and health insurance [14]. These are echoed in qualitative studies emerging in Southeast Asia, the United Kingdom, mainland Europe, and the United States [15–17], but research on groups in the Caribbean region specifically surrounding diabetic foot disease is lacking.

Section 4.2., paragraph 1 should read:

Unlike the situations in the United Kingdom and United States, where people of African descent are an ethnic minority, Afro-Caribbean people are the majority ethnic group in Barbados. The long-standing cultural distrust of medical systems and transgenerational preference for alternative treatments seem to be less linked to ethnic minority status and perhaps more to community experiences and preferences for natural or alternative therapies. This has been previously documented in studies reflecting on diabetic education [28], asthma, smoking and lung cancer [29].

The following reference has been removed:

28. Baiden, D.; Parry, M.; Nerenberg, K.; Hillan, E.M.; Dogba, M.J. Connecting the Dots: Structural Racism, Intersectionality, and Cardiovascular Health Outcomes for African, Caribbean, and Black Mothers. *Health Equity* **2022**, *6*, 402–405.

With this correction, the order of some references has been adjusted accordingly. The authors state that the scientific conclusions are unaffected. This correction was approved by the Academic Editor. The original publication has also been updated.
