# Supplementary material for: Multiple Lenses to Unearth Hidden Voices: Living with Diabetic Foot Ulceration in an Afro-Caribbean Community
Source: Int J Environ Res Public Health. 2025 Feb 18;22(2):304. doi: 10.3390/ijerph22020304 (PMC11854952; doi:10.3390/ijerph22020304)
Supplement: Supplementary file 1 [file ijerph-22-00304-s001.zip › ijerph-3398557-supplementary.pdf]

Supplementary Materials S1

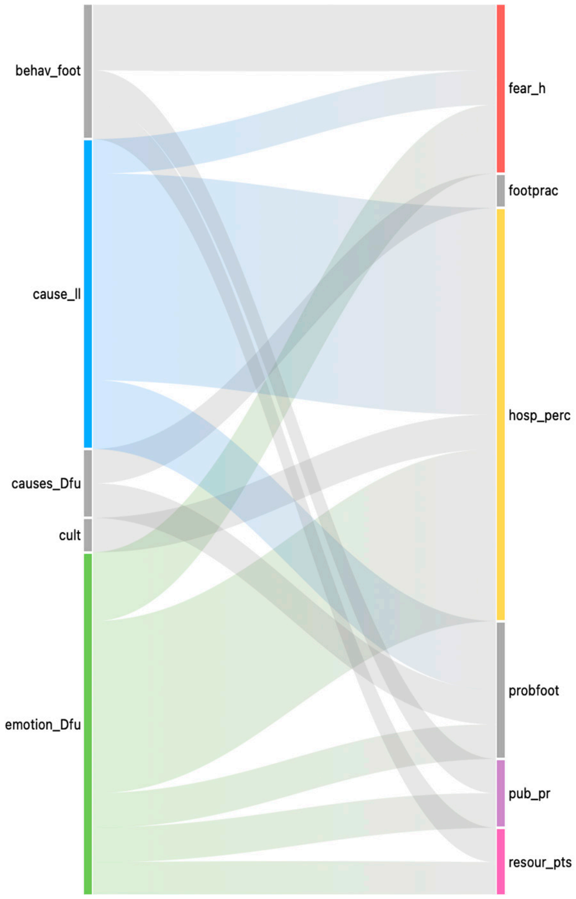

Code	Definition
behav_foot	Any reference to any behavior modification observed in patients with diabetic foot ulcer in Barbados
caus_ll	Any reference to causes of limb loss
causes_Dfu	Any reference to causes of diabetic foot ulcer
cult	References to the impact of culture on diabetic foot practices and education
emotion_Dfu	Any reference to the role of emotion in diabetic foot ulcer management
Fear_h	Any reference to patient fear of seeking care at the hospital or clinic
footprac	Any reference to experiences of local foot practices that contribute to diabetic foot ulcer
hosp_perc	Any reference to the perspective of the hospital in the role or treatment of diabetic foot ulcer
probfoot	Reference by healthcare practitioner to their perceptions or experiences OF dfu being a significant/ priority health condition in Barbados
pub_pr	References to differences in public and private healthcare systems
resour_pts	Any reference to challenge of social disadvantaged status in role in DFU

Figure S1. Sankey Coding Chart
